# Deformation Behavior of 3D Printed Auxetic Structures of Thermoplastic Polymers: PLA, PBAT, and Blends

**DOI:** 10.3390/polym15020389

**Published:** 2023-01-11

**Authors:** Jonas Hufert, Axel Grebhardt, Yanling Schneider, Christian Bonten, Siegfried Schmauder

**Affiliations:** 1 Institut für Kunststofftechnik, University of Stuttgart, Paffenwaldring 32, 70569 Stuttgart, Germany; 2 Institute for Materials Testing, Materials Science and Strength of Materials, University of Stuttgart, Pfaffenwaldring 32, 70569 Stuttgart, Germany

**Keywords:** auxetic structures, strand deposition process, Poisson’s ratio, filament extrusion, computed tomography

## Abstract

Auxetic structures have a negative Poisson’s ratio and therefore expand transversely to the direction of loading instead of tapering. This unique behavior is not caused by the materials used, but by the structure, and thus offers completely new functionalities and design possibilities. As a rule, auxetic structures have a very complex geometry, which makes cost-effective production possible only by means of additive manufacturing processes. Due to the high design freedom of the strand deposition method, it makes sense to manufacture auxetic structures using this process. Therefore, in this project, polylactide acid (PLA), polybutylene adipate terephthalate (PBAT), and blends of the two polymers were produced and characterized. Filaments of the two polymers and a blend were extruded, processed into auxetic structures by strand deposition process (SDP), and investigated for their properties, primarily their Poisson’s ratio. The Poisson’s ratio was determined and the influence of the material on it was identified. A specific number of 5 × 5 unit cells has been found to be ideal for investigation. Dual printed specimens showed a similar auxetic behavior as the specimens made of pure PBAT. Likewise, multiple loading and unloading of the structure is possible. Furthermore, in-situ computed tomography revealed the detailed characterization of the initial state, including the warpage of the structures, damage, and traced auxetic behavior in detail.

## 1. Introduction

**Figure 1:**
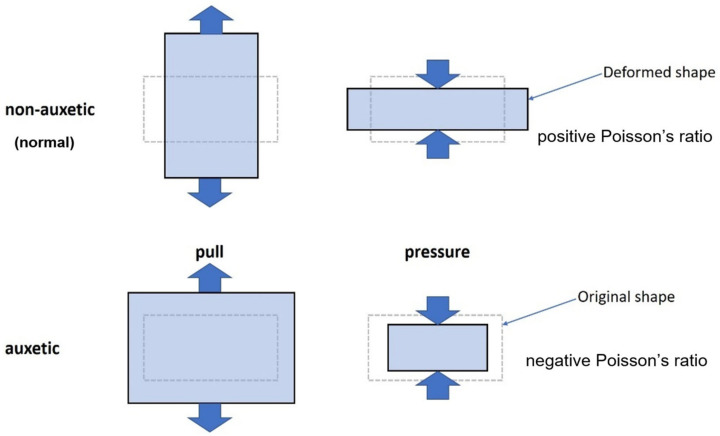
Comparison of conventional (positive Poisson’s ratio) and auxetic (negative Poisson’s ratio) deformation behavior.

The term “auxetic” describes an atypical deformation behavior of materials or structures. The term was coined by Evans [1] and describes a contrary transverse deformation behavior compared to conventional (non-auxetic) materials and structures. Under tensile loading, their cross-section increases and, under compressive loading, it decreases, as shown in Figure 1. This results in a negative transverse contraction number, or Poisson’s ratio 𝜈, as given in Equation (1), where *ε_x_* is the strain in loading direction and *ε_y_* is the strain transvers to the loading direction [2]. The Poisson’s ratio is defined as the negative ratio of the transverse strain to the longitudinal strain [3], meaning that the Poisson’s ratio for non-auxetic structures and materials remains greater than zero.

(1) $$v=\frac{-\varepsilon_{y}}{\varepsilon_{x}},$$

A non-auxetic structure under tensile load reacts by lengthening in the direction of load and shortening perpendicular to it, as already mentioned. In contrast, the same load on auxetic structures causes a lengthening in a perpendicular direction [4].

Due to the extraordinary deformation behavior, innovative design approaches can be realized to improve the properties of components, such as energy absorption properties or weight efficiency [5]. Auxetics are used in the fields of robotics, biomedicine, electronics, and acoustics, among others [6,7]. These structures are also used in the manufacturing of sandwich, composite, and lightweight components [4].

**Figure 2:**
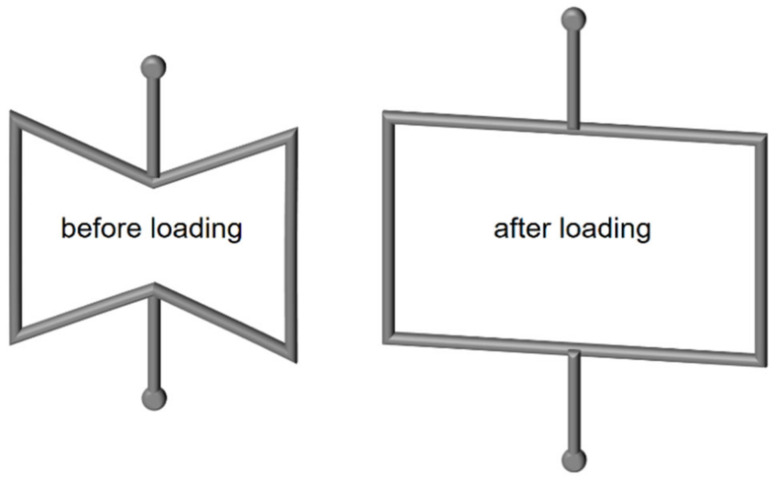
Re-entrant inverse honeycomb structure.

There are naturally occurring materials with auxetic properties, such as α-cristobalite, a silicon dioxide [8,9]. However, these are not considered in this work. The focus is on artificially produced auxetic structures using thermoplastic polymers. There is a wide variety of possible structures with auxetic behavior [10,11]. A simple model is a two-dimensional inverse honeycomb structure with re-entrant corners [12]. This structure is shown in Figure 2, and their tensioning handles are indicated with spots. In the current work, the term honeycomb is used instead of inverse honeycomb to ease readability.

**Figure 3:**
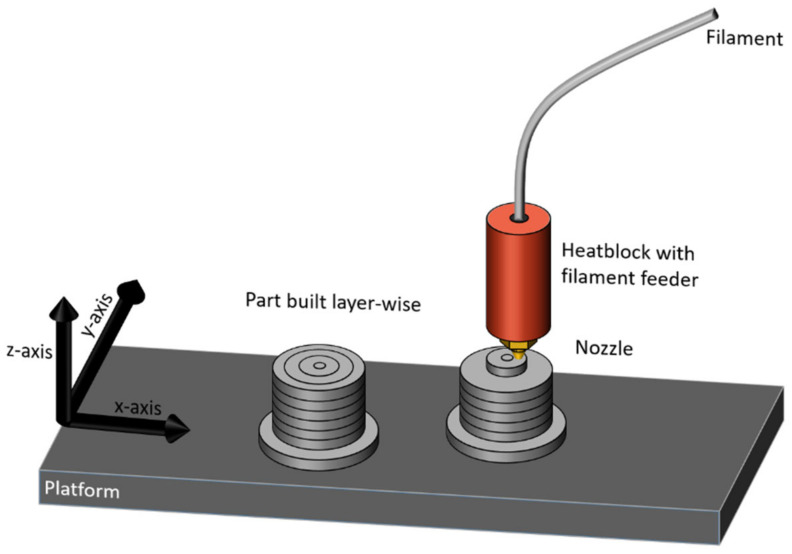
Schematic illustration of the SDP.

For the production of a two-dimensional honeycomb structure with recessed corners, the strand deposition process (SDP), also referred to as fused filament fabrication, is suitable due to its high design freedom. All geometry parameters of the structure can be varied without additional effort [13,14]. The strand deposition (SDP) is characterized by a layered structure of the component. Figure 3 schematically illustrates the principle of the SDP. A polymer filament is melted in a die and extruded through the nozzle. The melted strand is deposited layer-wise in the desired geometry in a line by means of a plotter mechanism in the *x*- and *y*-axes [15,16].

Polylactide acid (PLA) is a bio-based and biodegradable thermoplastic polymer with good processability [17] regarding SDP [18]. Polybutylene adipate terephthalate (PBAT) is a bio-based and biodegradable thermoplastic polymer as well; however, its deformation behavior differs from PLA [19]. Though the biodegradability of the blends of PLA and PBAT is not as good as that of the pure polymers [20], blends can be manufactured by using a coupling agent to ease chemical miscibility [21,22]. The printability of such blends has been investigated and proven, e.g., by Cardoso et al. [23]. Regarding the high potential of bio-based and biodegradable polymers, auxetic structures manufactured from these materials could be used in, e.g., healthcare applications.

This work aims to show the influence of the total number of honeycombs and the material on the transverse contraction of a two-dimensional honeycomb structure with re-entrant corners. It will be shown how different stiffnesses affect the deformation behavior of the structure and whether the transverse contraction number of the honeycomb structure can be modified by combining two materials. In a further step, computed tomography (CT) will show the warpage and the initial damage caused by cooling and the used manufacturing process. The CT test will also disclose the auxetic deformation behavior in detail.

## 2. Materials and Methods

### 2.1. Compounding of Materials and Filament Extrusion

#### 2.1.1. Materials

For the experiments, polylactide acid (PLA) 4032D (NatureWorks LLC, Minnetonka, MN, USA) was used [24]. This polymer is a completely bio-based and biodegradable polymer. PLA was selected because it provides very good printability in the SDP and possesses a relatively high stiffness. In addition to PLA, polybutylene adipate terephthalate (PBAT) Ecoflex F Blend C1200 (BASF, Ludwigshafen, Germany), a biodegradable polymer, was selected [25]. PBAT was chosen because of its low stiffness and high elongation. Furthermore, PBAT is a suitable blend partner for PLA. Blending PBAT with PLA allows for the modification of mechanical properties such as the stiffness and maximum elongation [19]. Besides PLA and PBAT, the blends of these two polymers were also prepared by adding small amounts of the coupling agent Joncryl ADR 4468 (BASF, Ludwigshafen, Germany) [26].

#### 2.1.2. Compounding

The compounding was carried out on an EBVP25 twin screw extruder (O.M.C., Saronno, Italy). The used polymers and the coupling agent were added gravimetrically and were always given as weight percent (wt%) in the following. The manufactured compounds and the polymers are listed in Table 1. Only that for Compound C2–C5 0.7 wt% of the coupling agent was used.

**Figure 4:**
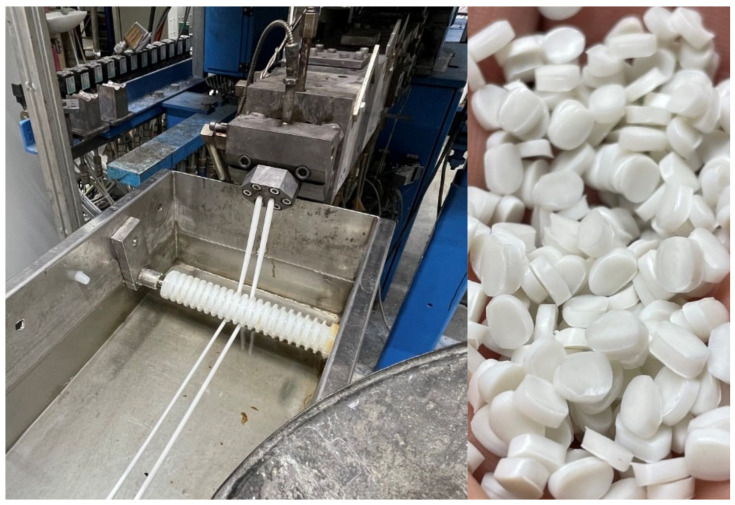
Compound C5 while compounding (**left**) and pellets (**right**).

The screw design of the compounding process included different kneading and mixing elements. In Figure 4, the extrusion of compound C5 is shown on the left and the pellets are shown on the right.

The temperature profile used is shown in Table 2.

#### 2.1.3. Filament Extrusion

A 30 × 25D single-screw extruder (COLLIN Lab & Pilot Solutions GmbH, Maitenbeth, Germany) was used for filament extrusion. The used temperature profile of the filament extrusion process is shown in Table 3. The filaments were extruded with a diameter of 1.75 mm at a speed of 25 1/min. The filament diameter was continuously determined and checked with an ODAC 15XY laser measuring device (Zumbach Electronic AG, Orpund, Switzerland).

### 2.2. Characterization of the Materials

Blending of the polymers was successfully carried out with the presented process parameters. Subsequently, filaments with a diameter of 1.75 mm were extruded from the polymers and each blend. The produced compounds were tested for their rheological properties using a Discovery HR2 plate-plate rheometer (TA Instruments, Newcastle, DE, USA) at a temperature of 180 °C. Measurements were carried out from 0.1 rad/s to 100 rad/s (frequency sweep mode) to provide information on the extrudability of the filaments and their printability. Furthermore, time sweeps were performed at a constant angular frequency of 10 rad/s and a temperature of 180 °C. The measurement duration was set to 2000 s.

To determine the mechanical properties, tensile tests were carried out on a universal testing machine (ZwickRoell, Ulm, Germany). The test speed was set to 5 mm/min for all tested specimens with a clamping length of 115 mm. For this purpose, tensile test specimens were produced on an Allrounder 370S 700-100/10 (Arburg, Lossburg, Germany).

**Figure 5:**
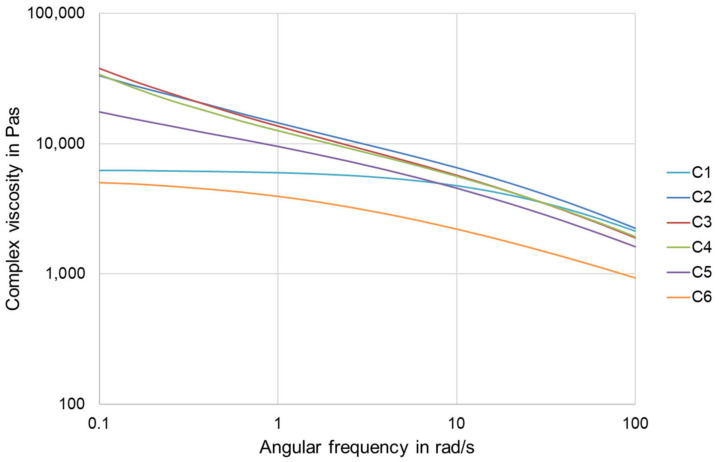
Complex viscosity of the compounds as a function of the angular frequency.

Figure 5 shows the flow behavior presented by the complex viscosity of the produced compounds as a function of the angular frequency. Compound C6 shows the lowest complex viscosity. The complex viscosity of polymer C1 to compound C5 is similar at higher angular frequencies. However, the viscosities of the compounds C2, C3, C4, and C5 do not show a Newtonian plateau at lower angular frequencies, yet the complex viscosity continues to increase.

**Figure 6:**
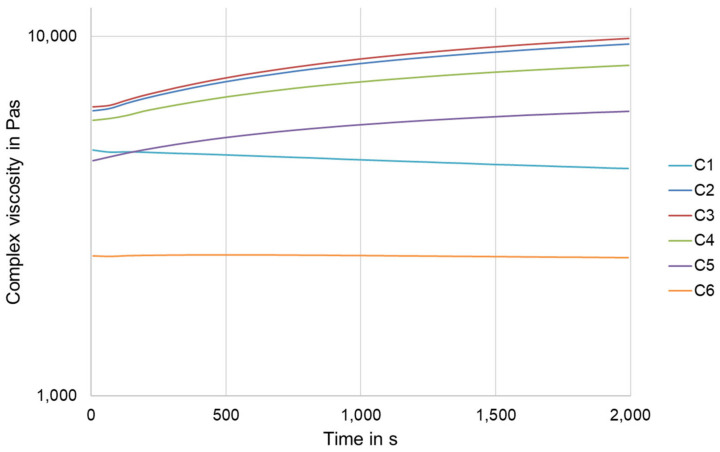
Complex viscosity of the compounds as a function of time.

Figure 6 shows the flow behavior of the produced compounds as a function of time. The viscosity of polymer C1 decreases over time. The decrease in viscosity can be explained by thermal degradation over time. For compound C6, no changes in viscosity can be observed over the entire measurement period. For the compounds C2, C3, C4, and C5, the viscosity increases over time. This can be explained by further reactions between the coupling agent and the blend partners. Presumably, not all functional groups have reacted during compounding, allowing chain extension to continue.

The characteristic values determined from the tensile tests for the compounds are shown in Table 4.

### 2.3. Fabrication of Auxetic Structures

Two-dimensional honeycomb structures with additional clamping jaws at the upper and lower ends were printed from the polymer C1, compound C5, and polymer C6 using the SDP. Two different printers were used to manufacture the auxetic structures. The structures from polymer C1 and the dual printed structures from two materials were 3D printed on a Raise3D Pro2 (Raise 3D Technologies, Inc., Irvine, CA, USA). The filaments from compound C5 and polymer C6 were processed into auxetic structures on a Prusa i3 MK2 (Prusa Research a.s., Prague, Czech Republic). Slicing was performed with the software ideaMaker (Raise 3D Technologies, Inc, Irvine, CA, USA). The most relevant process parameters used are listed in Table 5.

**Figure 7:**
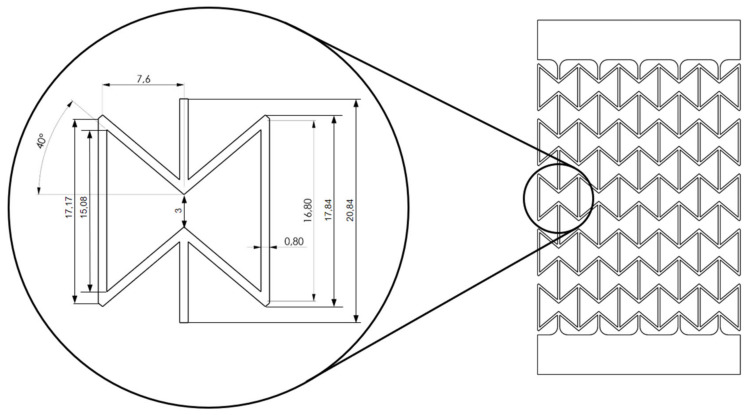
Schematic representation of a single honeycomb with its dimensions.

The honeycomb structure consists of individual combs, with the dimensions in Figure 7 given in millimeters.

**Figure 8:**
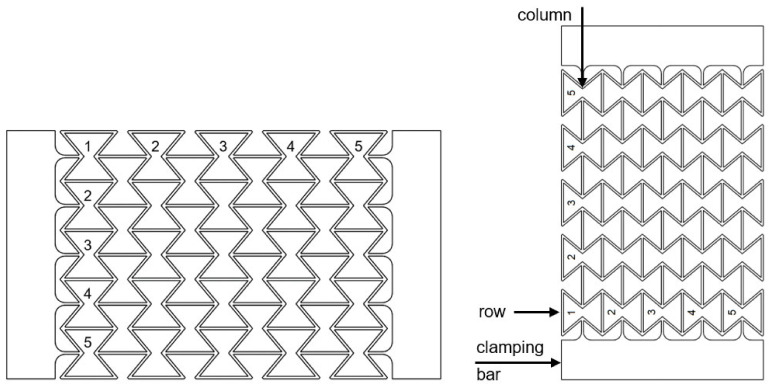
Schematic representation of the honeycomb structure used with the clamping jaws.

Structures with three, five, and seven honeycombs in longitudinal and transverse direction were 3D printed. Because of boundary issues, the characterization of the auxeticity of the structures consisting of three honeycombs is limited. Detailed information is discussed and, due to this, the structures of the three honeycombs are mentioned in this work but are not investigated in detail. A schematic representation of the structure with the five honeycombs in longitudinal and transverse direction and the clamping jaws for testing in a universal testing machine is shown in Figure 8. The test specimens were designated according to the compound count of the honeycomb’s principle. However, a honeycomb specimen from polymer C1 with five honeycombs in the longitudinal and transverse direction is thus designated C1_5.

**Figure 9:**
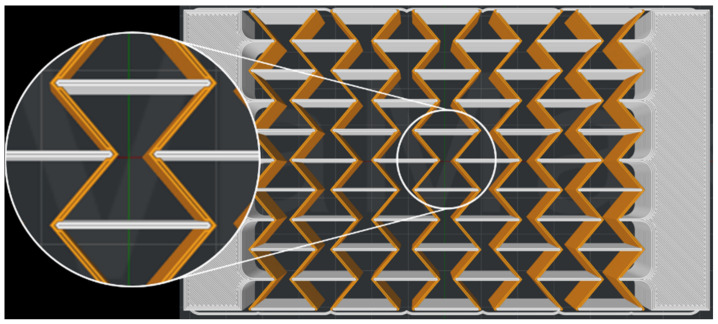
Schematic representation of a dual printed honeycomb structure.

In a second step, honeycomb structures were 3D printed by dual printing using the polymers C1 and C6. The bars in the longitudinal direction were printed from the stiffer polymer C1 and the bars in the transverse direction from the more ductile compound C6. This is shown schematically in Figure 9. Polymer C1 is shown in light gray and compound C6 in orange. The honeycombs made of two polymers are referred to C1_C6_5 in the following.

**Figure 10:**
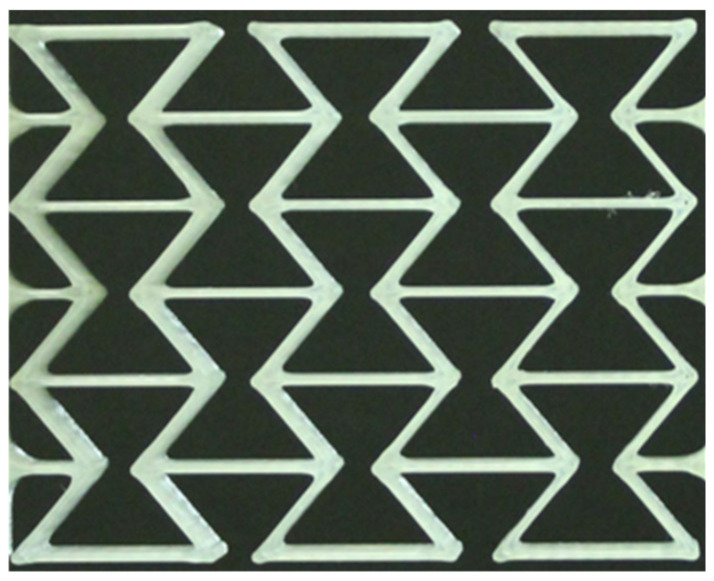
Auxetic structure with 3 × 3 honeycombs in longitudinal (horizontal) and transverse (vertical) direction.

The fabrication of auxetic structures with different numbers of honeycombs was possible with all compounds. Figure 10 shows an example of a honeycomb structure with three honeycombs in the longitudinal direction and three in the transverse direction after 3D printing from polymer C1, which, as mentioned, is referred to as C1_3. The clamping bars at the upper and lower ends of the structure are not visible in Figure 10.

### 2.4. Determination of the Poisson’s Ratio

**Figure 11:**
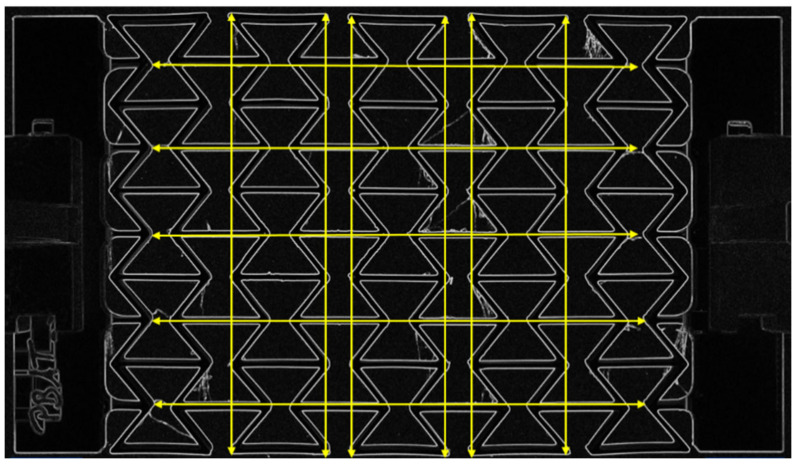
Exemplary representation of the measurement of an auxetic structure.

The tests to determine the Poisson’s ratio were carried out on the same universal testing machine as the tensile tests. The transverse contraction number was determined using images taken with an EOS 1300D single-lens reflex camera (Canon, Tokyo, Japan). For this purpose, the images were analyzed using the image processing software Fiji (ImageJ 1.53q, National Institutes of Health, Bethesda, MD, USA). The measurement of an auxetic structure is shown, for example, on a 5 × 5 honeycomb structure in Figure 11. The transverse contraction number was then determined via the longitudinal and transverse strain.

**Figure 12:**
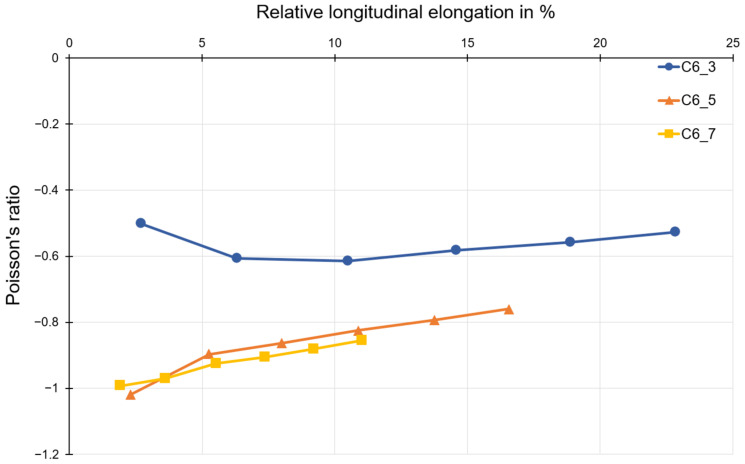
Poisson’s ratio as a function of relative longitudinal elongation for tested specimens made of polymer C6.

The influence of the number of honeycombs of the structure and the material on the deformation behavior can be shown using the investigation results. According to the theory, changing the number of honeycombs in the longitudinal and transverse directions by the equal amount does not change the Poisson’s ratio, since there is a proportional relationship. The results of the transverse contraction number for compound C6 is shown in Figure 12. The Poisson’s ratio increases with increasing strain for all test specimens. The Poisson’s ratio is in the range of about −1.0 to −0.8 for structures with a honeycomb number of five and seven, respectively. The structure with a honeycomb number of three, however, shows minor auxetic behavior, with a Poisson’s ratio of about −0.6.

**Figure 13:**
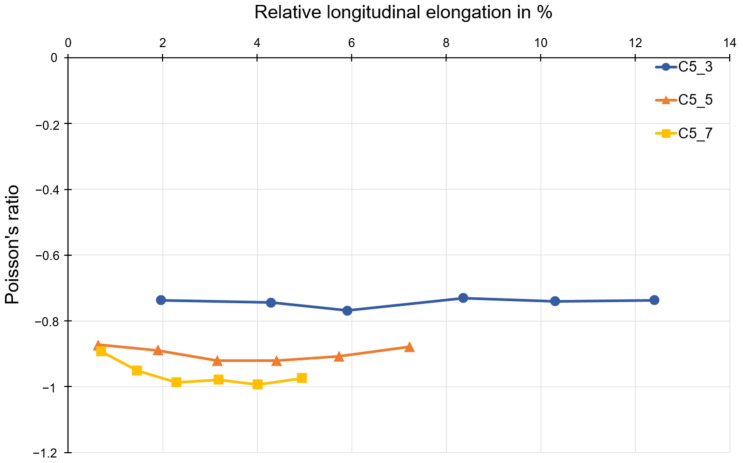
Poisson’s ratio as a function of longitudinal strain for test specimens made of compound C5.

Figure 13 shows the Poisson’s ratios determined for compound C5. For this material, an almost constant Poisson’s ratio can be observed over the complete longitudinal strain range. For compound C5, the Poisson’s ratio is about −1.0 to −0.9 for structures with five and seven honeycombs, respectively. This corresponds to the value range of polymer C6 with five and seven honeycombs.

**Figure 14:**
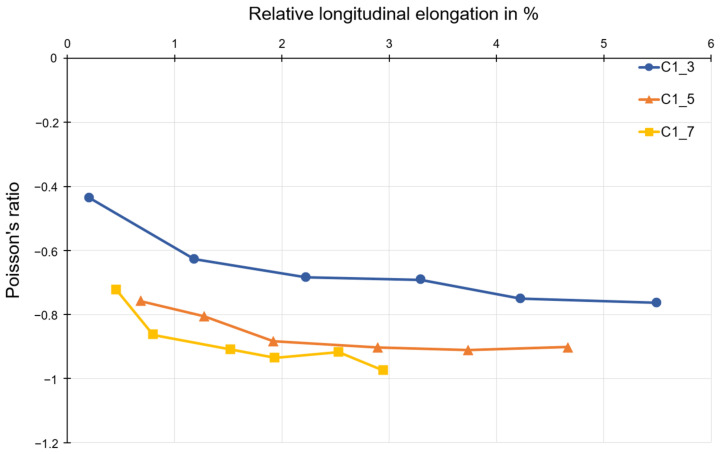
Poisson’s ratio as a function of longitudinal strain for test specimens made of polymer C1.

For polymer C1, as shown in Figure 14, the Poisson’s ratio decreases with increasing longitudinal elongation. Poisson’s ratios in the range of about −0.8 to −1.0 are also obtained for polymer C1, as for polymer C6 and compound C5.

**Figure 15:**
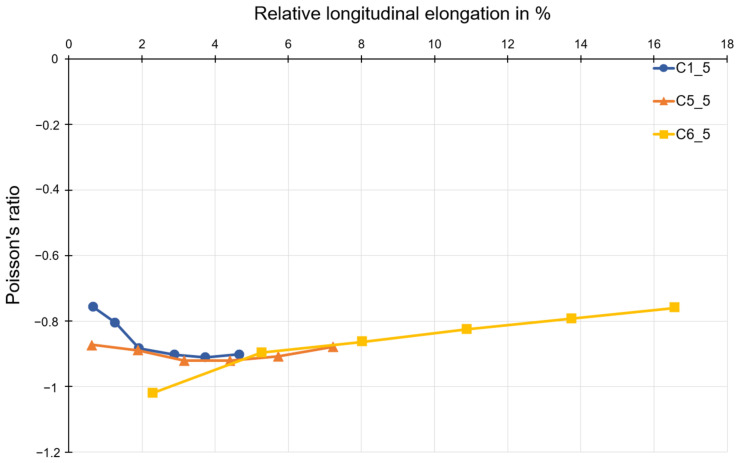
Poisson’s ratio as a function of longitudinal strain for test specimens with 5 × 5 honeycombs.

To evaluate the material influence, the results of all materials of a structure consisting of five honeycombs were plotted in a graph as a function of longitudinal strain. This is shown in Figure 15. There is a very good correlation between the curves if the first two measuring points of C1_5 and the first measuring point of C5_5 are neglected. If the structure is subjected to a tensile load, the Poisson’s ratio initially decreases, as already observed for polymer C1, until the minimum of the Poisson’s ratio is reached, at about 4 % relative longitudinal strain. After exceeding the minimum, an almost linear increase follows until the end of the load range.

**Figure 16:**
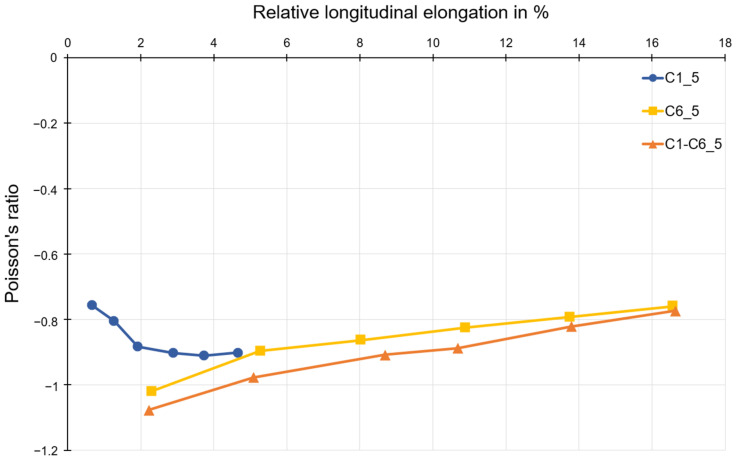
Comparison of Poisson’s ratio as a function of longitudinal elongation for dual printed specimens and specimens made of only one material.

To evaluate whether the Young’s modulus of the longitudinal crosspieces is influencing the course of Poisson’s ratio while elongated, dual printed specimens were examined. The route of Poisson’s ratio is not influenced by the Young’s modulus of the longitudinal crosspieces as shown in Figure 16. However, it is evident that the Poisson’s ratio of the dual printed specimens is slightly higher compared to the ones printed with polymer C6 only due to the higher stiffness of polymer C1 that is utilized for the longitudinal crosspieces. As a result, the transvers expansion of the dual printed specimens is increased at the same amount as the longitudinal elongation.

**Figure 17:**
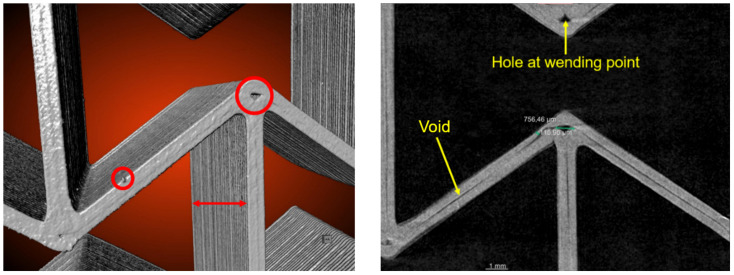
In-situ CT test detected imperfections in a 5 × 5 PLA sample at 0 mm loading (initial) state: bead, throughout holes, ribs (**left**) and local voids (**right**).

Figure 17 (left), obtained from a reconstruction of the computed tomography images for PLA, illustrates a detected bead, indicated by a small circle. In addition, holes are indicated by a large circle, and non-optimally placed layers are indicated by a double-headed arrow. The holes, mostly corresponding to the thickness of the crosspieces (Figure 17, right), locate at wending points of the printing path or the joining part of the inclined walls. Long and narrow voids (Figure 17, right), mostly with a short extension in the third direction, are also detected.

**Figure 18:**
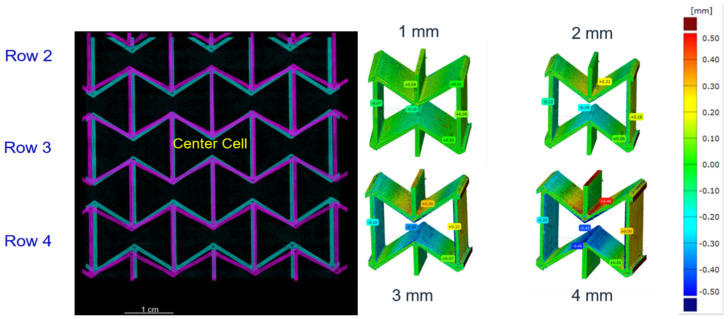
Auxetic deformation behavior of a 5 × 5 PLA sample: overlapping of the sample state at loadings of 0 mm (cyan) and 4 mm (magenta) loading (**left**); the measured local displacement of the center honeycomb using 2D-DIC (**right**).

During the in-situ CT tensile test, it was observed that the honeycombs in the middle row deformed less than the other rows. To visualize this, Figure 18 (left) presents the overlapping view of the measured structural state for the three middle rows for PLA at 0 mm (cyan) and 4 mm (magenta) loading displacement. From Figure 18, it can be traced that the center honeycomb possesses the least displacement of all. With the application of 2D-DIC, an inhomogeneous strain distribution is shown in Figure 18 (right). Thus, the local displacement distribution for the center honeycomb at 0–4 mm loading with a distance of 1 mm between measurements is demonstrated.

**Figure 19:**
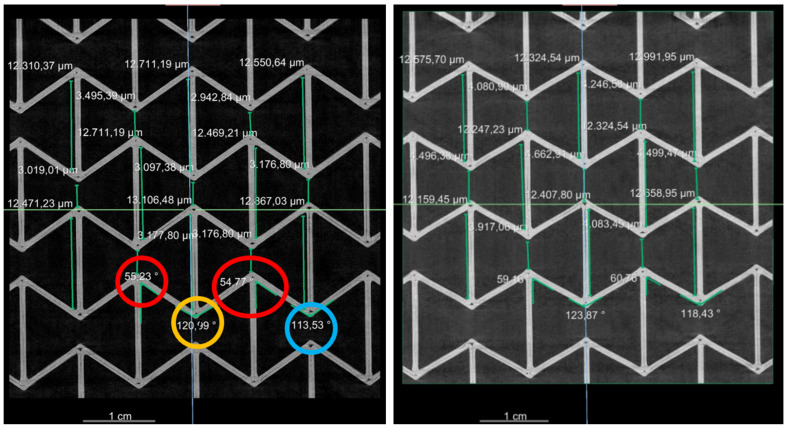
Measured wall length, wending points’ distance, and angles in individual honeycomb in a 5 × 5 PLA sample at 0 mm (**left**) and 4 mm (**right**) loading state in the same cut-out view as given in Figure 18. The angles marked in the red circle and ellipse are about 5° larger than the design value, while the ones marked in the yellow and blue circles are about 10°–20° larger.

**Figure 20:**
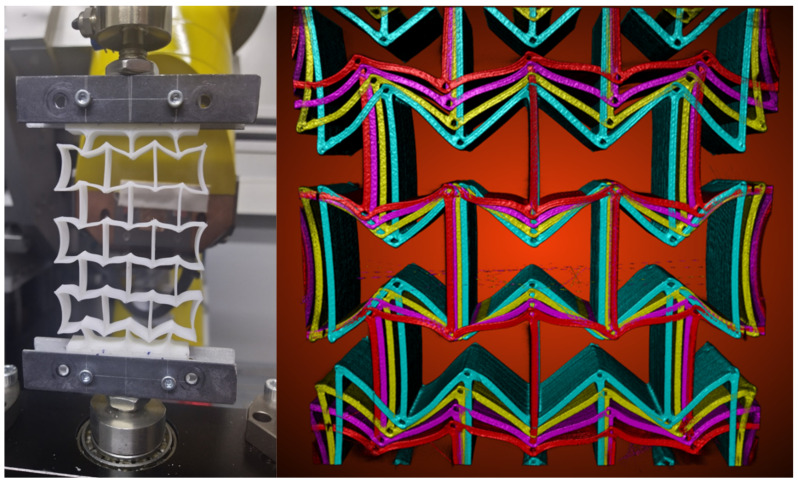
Deformed state of a 3 × 3 PBAT sample (**left**) and the overlapping view of the sample at loading state of 0 mm, 5 mm, 10 mm, and 15 mm presented with cyan, yellow, magenta, and red color, respectively (**right**).

**Figure 21:**
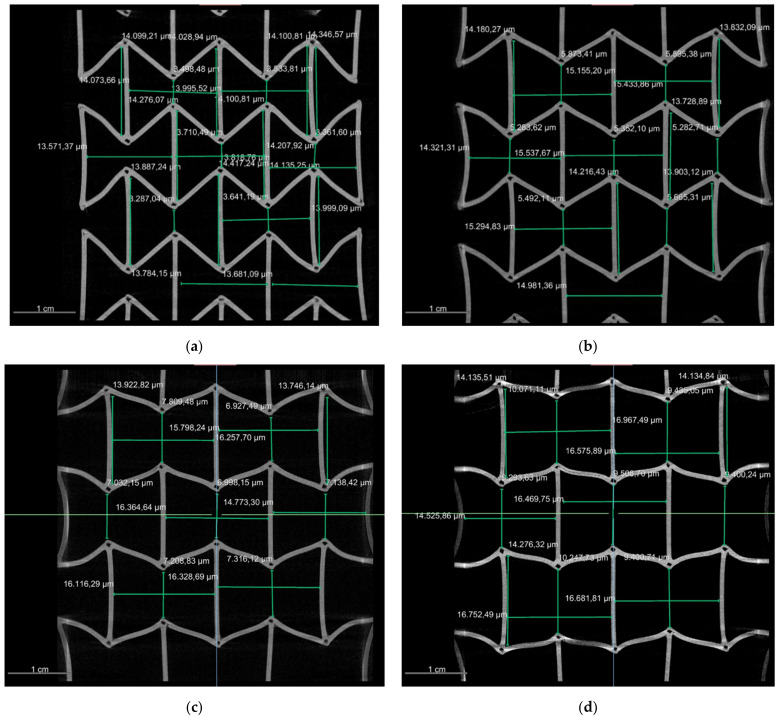
Measured wall length, wending points’ distance in individual honeycomb in a 3 × 3 PBAT sample at (**a**) 0 mm, (**b**) 5 mm, (**c**) 10 mm, and (**d**) 15 mm loading state.

Figure 19 presents the measured values of the individual wall lengths, the distance between two wending points, and the angles between two inclined walls for PLA. Concerning PBAT, a similar CT test was performed on a sample with 3 × 3 honeycombs. The reason for using 3 × 3 honeycombs instead of 5 × 5 is mentioned in Section 2.5. Figure 20 (left) shows the deformed auxetic structure made from PBAT. Figure 20 (right), for PBAT, is similar to Figure 18 (left) for PLA, showing an overlapped deformed state. The loadings are 0–15 mm for Figure 20 (right), with a distance of 5 mm between the measurements. The deformed structure is presented in cyan, yellow, magenta, and red for the loading amounts mentioned above, respectively. Quantified values in the deformed PBAT sample are shown in Figure 21 for the honeycomb wall length, the neighboring walls’ distance, and the distance between the wending points of two inclined walls. It covers the same loading state as in Figure 20 (right).

### 2.5. In-Situ Computed Tomography (CT) Test

The auxetic structural behavior measured by the conventional tensile testing machine illustrated a large deformation discrepancy between the upper as well as the lower rows and the middle three rows of the honeycombs. To achieve quantitative data for the analysis, in-situ CT tests with a combination of digital image correlation (DIC) are preferred, which allows for the calculation of the local displacement/strain maps [27,28,29,30]. A more detailed presentation of the CT test and DIC technique is shown in [27,28,29,30]. Furthermore, the tomographic scan can reveal the initial damage and the warpage caused by the 3D printing and the cooling process, respectively.

**Figure 22:**
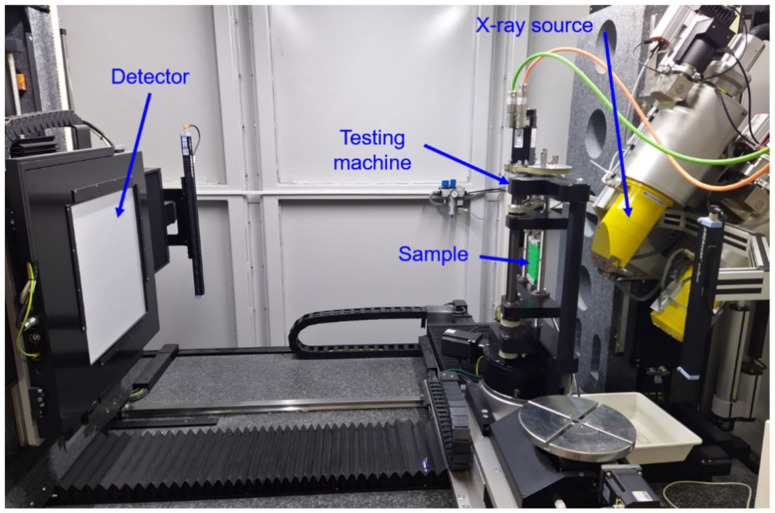
In-situ CT equipment (RIF e. V. Dortmund, Germany).

Figure 22 shows the in-situ CT equipment (RIF e. V. Dortmund, Germany) used for the measurement mentioned above. The tests for PLA and PBAT have been carried out with the v|tome|x L 240 (GE Sensing & Inspection Technologies GmbH, Wunstorf, Germany). For PLA, the test parameters were set to 80 KeV and 100 µA. The voxel size is 45 µm. For PBAT, the test parameter is 120 KeV and 80 µA with a voxel size of 25 µm. The PLA sample consists of 5 × 5 honeycombs, while PBAT consists of 3 × 3 due to the limited vertical space of the testing rig and PBAT’s extra high tensile deformation ability. During the tensile test, five scans were performed for PLA at 0, 1, 2, 3, and 4 mm loading magnitude and four for PBAT, at 0, 5, 10, and 15 mm.

## 3. Discussion

The results of the rheological investigations show that the viscosity of the compounds is in a range that is well suited for processing by SDP. This observation is confirmed as compound preparation and further processing into filaments was performed without difficulty, such as, e.g., in [21,22]. In addition, the results suggest that the filaments from the prepared compounds (C2, C3, C4, and C5) exhibit similar printing behavior as the filaments from pure PLA (polymer C1). The filaments made from pure PBAT (polymer C6) require a slightly different control process during printing than those made from pure PLA due to their lower viscosity. This is evident at the lower nozzle temperature of 200 °C compared to 220 °C for all other filaments.

The results from the tensile tests show that it is possible to adjust the Young’s modulus, tensile strength, and elongation at break by blending PLA with PBAT, which was also found in [19]. The Young’s modulus and tensile strength decrease significantly with an increasing proportion of PBAT in the compounds and approach the values of pure PBAT. In contrast, the determined value for the elongation at break increases with increasing PBAT content and seems to reach a plateau at about 170 MPa. The tensile test specimens made of pure PBAT did not break, as they reached the maximum measurable elongation of 350%. Compounding is therefore a good way of specifically adapting mechanical material properties, as mentioned in [19]. This allows the deformation range of subsequent auxetic structures to be adjusted in a targeted manner.

Auxetic structures were produced from the polymer C1, compound C5, and polymer C6. An evaluation of the Poisson’s ratio for these three materials was also possible. Looking at the Poisson’s ratio curves for different numbers of honeycombs, it can be seen that the specimen with three honeycombs has a significantly higher Poisson’s ratio than −1 (mathematic values). That means that the auxetic behavior of structures consisting of three honeycombs is less than the one consisting of five or seven honeycombs, since the Poisson’s ratio near to −1 is accepted as optimal. In the current work, the discussion is excluded for the Poisson’s ratio lower than −1. In such cases, the lateral strain is higher than the loading direction, which is outside of the research goal of the project. The deviation that occurs can mainly be attributed to the type of restraint, since the restraint impedes the transverse strain in the first row below or above the restraint jaws. This leads to a distorted result for the Poisson’s ratio. For this reason, the results for structures with three honeycombs are not discussed. However, the results for structures with five and seven honeycombs can be compared and discussed. The results shown for polymer C1, compound C5, and polymer C6 allow for the assumption that the Poisson’s ratio does not change for the same number of honeycombs in the longitudinal and transverse directions. This is also consistent with the theoretical consideration of the transverse contraction number for a honeycomb structure with re-entrant corners.

However, the previous observation does not yet allow for any statement on the influence of the material on the Poisson’s ratio. For this reason, the results of all three compounds of a structure with five honeycombs were plotted in a diagram as a function of the longitudinal strain in Figure 15. It was found that the superimposed curves show the trend of the Poisson’s ratio independently of the compound-specific load limits. Each material represents the behavior in the respective deformation range. By choosing a material, the strain range of the resulting auxetic structure can be determined.

Due to the honeycomb geometry, the transverse strain should reach a maximum at the beginning of loading, since the honeycomb angle 𝛼 is smaller than 45°. Due to the folding out of the honeycomb webs, the angle decreases further. Accordingly, with further tensile loading, the increase in transverse strain decreases while the longitudinal strain continues to increase at a constant rate. This results in an increase in the Poisson’s ratio, as can be observed in compound C6 and also to some extent in C4. It is striking that this increase in Poisson’s ratio is not observed in polymer C1. Here, the Poisson’s ratio decreases over the entire deformation range. The deviation from this described course increases more and more with the increasing stiffness of the material. This suggests that a high stiffness of the material, especially at the nodal points, has an additional influence on the auxetic deformation behavior of the structure. Whether this is due to the design of the test specimens or due to the structure itself is the subject of further investigation.

The additive manufacturing process causes the holes at wending points (Figure 17) since the nozzle has some thickness, and two printing paths join each other at these wending points. Still, the viscosity stops dispersing the printed material to these wending points. As a result, such zones are not fully filled with material. The layer-by-layer printing process causes the ribs. Some thicker ribs appear at a regular distance and are possibly printing parameter-dependent. The thin and long voids should be the gaps between different printing lines in the same layer since the whole ribbon’s thickness is 0.8 mm and the printing thickness of a single path is 0.4 mm. The problem may be that the previously printed material is already cooled down and cannot provide enough adhesion to achieve a good connection with the currently printed material. Such voids are primarily short in the third direction.

Regarding the auxetic structural deformation behavior, it is reasonable that the honeycombs in the middle row (A-type) deform less than the others (Figure 18, left) since honeycombs in the rows near the loading sites (B-type) will firstly deform to reach geometrical compatibility. Then, the deformed B-type honeycombs will pull their neighboring honeycombs, and the A-type honeycombs will be pulled at last. In such a deformation process, the B-type honeycombs are influenced by the boundary conditions and will show more severe deformation than other honeycombs. A-type honeycombs in the middle row will show less deformation than other honeycombs. The displacement inside one honeycomb is inhomogeneous, still partly under tension and partly under compression (Figure 18, right). Experimental local displacement distribution will be used to calibrate the result from the finite element (FE) simulation, which will be presented in our consecutive works. Such calibration will also apply to the measured data in Figure 19. The comparison between the data in Figure 19 (left, as initial state) and the computer-aided design (CAD) data (Figure 7) illustrates the printing quality. The angles marked in the red circle and ellipse are about 5° larger than the design value, while the ones marked in the yellow and blue circles are about 10°–20°. However, in such a case, the half (5°–10°) should be considered as the discrepancy since they present the sum of two angles. Two significant aspects, the printing technique and the warpage due to cooling after printing, may cause a discrepancy between the CAD design and the real sample. A detailed evaluation of all the CT testing data, as shown in Figure 19, requires autonomous data processing such as machine learning or other algorithms. Such a result will be shown in our further works.

The CT measurement presents the same result for PBAT as for PLA, specifically that the A-type honeycombs deform less than the B-type ones. This is clearly presented in Figure 20 (right). PBAT has a high ductility but relatively low strength. This property might lead to warpage while cooling down each layer during 3D printing. The used sample (A-type) in Figure 21a shows relatively large warpage compared to the designated geometry for the outer walls as marked in rectangles. A-type also possesses a more considerable warpage than others, which means that further CT tests have to be performed to gather more data and to improve the printing quality. The maximum load applied on a PBAT auxetic structure introduces approximately the deformed state, as shown in Figure 21d, since the auxetic potential is nearly used up at this deformed state.

## 4. Conclusions

The current work investigates the deformation behavior of additively manufactured auxetic structures composed of the re-entrant honeycombs (inverse honeycomb) of different PLA and PBAT compounds. One of the main indicating factors for such an auxetic performance is the Poisson’s ratio. The total number of honeycombs in the structure also influences the deformation behavior. Auxetic structures with various honeycombs are deformed in tension to obtain the optimum number of honeycombs. Detailed auxetic deformation behavior is realized by in-situ CT tests with the combination of the DIC technique.

In conclusion, all materials were processed without restrictions by means of the strand deposition process (SDP). A determination of the Poisson’s ratio was possible with the manufactured test specimens. The structures with three honeycombs each in longitudinal and transverse direction did not provide representative results. This is due to the influences of the restraint on the deformation behavior, which was too large. The auxetic structure with 5 × 5 honeycombs has been shown to be the optimal one compared to those with 3 × 3 and 7 × 7 honeycombs. Both structures with 5 × 5 and 7 × 7 honeycombs illustrate Poisson’s ratios near the optimum value –1 at different loading state. The investigations have shown that neither the number of honeycombs nor the material noticeably influences the magnitude of the Poisson’s ratio of an auxetic honeycomb structure. However, the mechanical properties of the material used, in particular, the stiffness, determines the course of the Poisson’s ratio with increasing strain. Depending on the requirements, the Poisson’s ratio can be adapted to the demands of a use case by selecting a suitable material.

It is advantageous to improve the printing quality to compare the CAD geometry and the measured initial structural state visualized by CT tomography. The test results of a PLA sample illustrate a deviation of 5°–10° for the angles in honeycombs in this work. The honeycombs in the middle row deform less than in other rows. Honeycombs in the upper and lower rows experience less deformation due to severe boundary conditions, which means such honeycombs should be excluded from the calculation of structural Poisson’s ratio. The local displacement and strain distribution is achieved based on tomographic images and DIC data. Such deduced testing results can calibrate the results of FE simulations, which will be shown in our consecutive works.

## Figures and Tables

**Table 1:** Materials and composition.

Compound	PLA wt%	PBAT wt%
C1	100	0
C2	80	20
C3	70	30
C4	60	40
C5	40	60
C6	0	100

**Table 2:** Temperature profile during compounding.

Compound	Zone	1	2	3	4	5	6	7	8	9	10	11
C1, C2, C3, C4	Temperature in °C	30	80	120	160	185	185	185	185	185	195	210
C5, C6		30	80	120	160	185	185	185	185	185	195	200

**Table 3:** Temperature profile during filament extrusion.

Compound	Zone	1	2	3	4	5
C1, C2, C3, C4, C5	Temperature in °C	160	190	205	205	190
C6		160	180	185	185	185

**Table 4:** Determined mechanical properties of the compounds.

Name	Young’s Modulus in MPa	Tensile Strength in MPa	Elongation at Break in %
C1	3813 ± 192	63 ± 3	3.5 ± 1.3
C2	2682 ± 139	49 ± 3	20 ± 16
C3	1883 ± 189	29 ± 3	182 ± 45
C4	1459 ± 75	28 ± 1	160 ± 17
C5	485 ± 46	20 ± 1	176 ± 9
C6	86 ± 2	15 ± 1	>350

**Table 5:** Process parameters for SDP.

Compound	C1	C5	C6
Layer height in mm	0.20	0.20	0.20
Extrusion width in mm	0.40	0.44	0.44
Extruder temperature in °C	210	220	200
Printing speed in mm/s	45	35	35
Printing speed first layer in mm/s	13	17	17
Heated bed temperature in °C	50	50	60

## Data Availability

Not applicable.
